# Hospital Administration

**Published:** 1898-07-09

**Authors:** 


### HOSPITAL ADMINISTRATION.

### A SANITARY QUESTION AT THE SALISBURY INFIRMARY.

A somewhat curious problem has been set before us. It appears that the sanitary authorities at Salisbury have decided to erect a public urinal for both sexes, and have chosen for its site a point in the public street opposite the Salisbury Infirmary, almost immediately in front of the children's and the ovariotomy wards. This course seemed to be so objectionable that a section of the Infirmary Committee were in favour of offering the sanitary authority a site actually in the infirmary grounds, which, although really nearer to the institution, could be well screened by trees. We are asked our opinion on the matter. Now what are we to answer to such a question?

In the first place it is quite clear that the perception of what a hospital is and ought to be has not yet permeated the minds of the local authorities who are responsible for the proposition to erect a urinal for both sexes immediately opposite the General Hospital at Salisbury. A hospital is a place which should be made as attractive and as health-restoring as possible. This proposal at Salisbury would tend to degrade the hospital, to make many people hesitate to subscribe to it, and at the same time would prove a nuisance directly by rendering it necessary to obscure the glass of the windows of the wards overlooking this public convenience, and indirectly on sanitary grounds. We say on sanitary grounds, because our experience of public conveniences of this kind is that they are never properly looked after, and that they naturally tend to increase as nuisances year by year. The medical staff have done well to protest against any portion of the infirmary site being utilised for the erection of a urinal, but they will do better still if they combine to arouse public opinion in Salisbury to the injury and wrong which the local authorities propose to do to this hospital, and to insist that no urinal shall be erected opposite to the infirmary. They might go further now that the local authorities have become aggressive in this matter, and insist upon the removal of the cab-rank, which, under no circumstances, ought to be placed immediately opposite a public hospital. The noise and litter and impurity arising from a cab-rank should cause local opinion, on the ground of humanity alone, to insist upon its removal.

We agree with the medical staff, who have raised a protest against the proposed building being placed within the infirmary grounds. It is a very serious matter for a board to part with land which they hold in trust for certain purposes, however desirable it may seem for other purposes, and we are glad to find that by a large majority the governors have declined to entertain the proposal. Whether, however, it is right and proper that such a structure should be erected opposite the infirmary is another matter, and depends much on local circumstances. There can be but little doubt that such a latrine, unless it is very well managed, *i.e.*, unless it is under the constant supervision of a caretaker and provided with frequently-acting flush tanks, is sure to be a nuisance. If, however, it is properly managed, we are not prepared to say that it would of necessity be injurious if placed in the position proposed: it certainly need not be as offensive as a cab-rank, and we believe that a cab-rank already exists at the point in question.

We do not pretend to know the hidden springs of local action in this matter, but we cannot avoid wondering why, of all parts of the city, this particular spot should be chosen for such a purpose; nor can we see why the sanitary authority should have come to a decision on the matter without making any previous communication to the Infirmary Committee, unless, indeed, the corporation had its eye on Naboth's vineyard, and thought to force the hand of the Infirmary Committee, and get them to offer in exchange the coveted bit of infirmary land by deciding offhand to erect a urinal in a position which was known to be objectionable from an infirmary point of view. Whatever are the motives at work, and however slight may be the risk of nuisance from a well-managed public convenience there can be no doubt that the governors of the infirmary are doing the proper thing in protesting at the earliest possible moment against anything which may even seem to endanger the health of the patients in their care.

